# Biologic Complexity in Sickle Cell Disease: Implications for Developing Targeted Therapeutics

**DOI:** 10.1155/2013/694146

**Published:** 2013-03-25

**Authors:** Beatrice E. Gee

**Affiliations:** Department of Pediatrics, Cardiovascular Research Institute, Morehouse School of Medicine, 720 Westview Drive SW, Atlanta, GA 30310-1495, USA

## Abstract

Current therapy for sickle cell disease (SCD) is limited to supportive treatment of complications, red blood cell transfusions, hydroxyurea, and stem cell transplantation. Difficulty in the translation of mechanistically based therapies may be the result of a reductionist approach focused on individual pathways, without having demonstrated their relative contribution to SCD complications. Many pathophysiologic processes in SCD are likely to interact simultaneously to contribute to acute vaso-occlusion or chronic vasculopathy. Applying concepts of systems biology and network medicine, models were developed to show relationships between the primary defect of sickle hemoglobin (Hb S) polymerization and the outcomes of acute pain and chronic vasculopathy. Pathophysiologic processes such as inflammation and oxidative stress are downstream by-products of Hb S polymerization, transduced through secondary pathways of hemolysis and vaso-occlusion. Pain, a common clinical trials endpoint, is also complex and may be influenced by factors outside of sickle cell polymerization and vascular occlusion. Future sickle cell research needs to better address the biologic complexity of both sickle cell disease and pain. The relevance of individual pathways to important sickle cell outcomes needs to be demonstrated *in vivo* before investing in expensive and labor-intensive clinical trials.

## 1. Introduction

Sickle cell disease (SCD) is a group of disorders caused by a mutation in the sequence of beta globin, leading to polymerized hemoglobin (sickle hemoglobin, hemoglobin S), hemolytic anemia, painful vaso-occlusive events, vascular remodeling, acute and chronic organ injury, and shortened lifespan. Sickle cell disease affects over 70,000 individuals in the United States, and there are at least 75,000 hospitalizations costing over $500 million annually for treatment of SCD complications [1]. While survival has greatly improved, the average lifespan for people with hemoglobin SS was estimated in 1994 to be in the midforties [2], significantly less than the average American. Despite well-described genetic and biochemical properties of sickle hemoglobin and many basic science discoveries about sickle cell pathophysiology, modern-day therapy continues to be limited to symptomatic treatment of pain, oxygen supplementation, antibiotics, red blood cell transfusions, and hydroxyurea. Hydroxyurea is an agent that induces fetal hemoglobin production and is the only drug approved for adults by the United States Food and Drug Administration that directly affects sickle cell outcomes. Stem cell transplantation from a histocompatible donor has a high cure rate, but many patients do not have a suitable donor. 

 Since the passing of the National Sickle Cell Control Act in 1972, over one billion dollars have been allocated from the National Heart, Lung and Blood Institute of the National Institutes of Health (NIH) for SCD research [3]. This funding has resulted in a significant body of research on SCD. The United States National Library of Medicine website lists over 7000 articles since 1950 meeting the search terms of “sickle cell research;” 482 are human clinical trials. As of December 1, 2012, the website http://www.clinicaltrials.gov/ showed 96 open intervention trials in sickle cell disease.  Table 1 shows the most common types of studies. Some of these emerging therapies have been recently reviewed [4].

 Director of the Division of Blood Diseases and Resources at the NIH, W. Keith Hoots, recently wrote, “Research over the decades indicates that the primary defect in hemoglobin that results in polymerization of the protein under low oxygen conditions and resultant cellular deformity of the red blood cell initiates a complex downstream pathogenesis associated with vascular injury, and organ ischemia. Deciphering this in a manner that informs successful therapies that improve all target organs continues to challenge hematologists” [5]. It is likely that this complexity is a barrier to successful translation of basic science discoveries in SCD to effective therapeutics. Sickle hemoglobin polymerization is associated with many abnormal downstream processes, but no single pathway has been shown to play a *primary or critical* role in complications occurring in people with SCD. Most mechanistically based clinical interventions have been designed to target individual pathways, but there is likely to be ongoing interaction within the body between different processes, so that even if one pathway is successfully blocked, others may still be active and continue to promote vaso-occlusion or other complications. To address biologic complexity in SCD, this paper will analyze examples of promising clinical trials that did not yield expected benefits, contrast reductionism with systems biology, present models that facilitate the visualization of interactions of mechanisms in SCD complications, and then discuss implications for future research.

## 2. Unexpected Outcomes of Promising Clinical Trials

 Preclinical studies and clinical trials targeting three different sickle cell pathways will be reviewed, including inhibition of adhesion by poloxamer 188, inhibition of Gardos channel-induced erythrocyte dehydration by senicapoc (ICA-17043), and treatment of acute pain episodes with inhaled nitric oxide. Comprehensive reviews of approaches to sickle cell treatment have been published elsewhere [4, 6, 7].

### 2.1. Antiadhesion Therapy with Poloxamer 188

 Fluorocarbon emulsions, including identical but variously named compounds Pluronic F-68, Flocor, RheoThRx, and poloxamer 188, have been studied in SCD since 1975 [8]. Pluronic F-68 was demonstrated to reduce *in vitro* sickle red cell static rigidity (stiffness), filterability through a 5 micron filter, and abolish adherence to endothelial monolayers [9]. It is believed to bind nonspecifically to the red cell membrane, “lubricating” the cells and “providing a hydrated, poorly compressible barrier that appears to block hydrophobic adhesive interactions (cell-cell, cell-protein, and protein-protein) in the blood [10].” Preclinical studies demonstrated “a reduction in blood viscosity, erythrocyte aggregation, adhesion to vascular endothelium, and an improvement in microvascular blood flow.” 

 A phase II randomized, double-blinded placebo-controlled trial (RCT) testing poloxamer 188 was conducted with fifty subjects with SCD who presented within 4–18 hours of onset of acute pain. They were treated with either placebo or poloxamer 188 infusion of 300 mg/kg for 60 minutes, followed by 47-hour maintenance infusion of 30 mg/kg/hr. In the 31 subjects who completed the 48 hour infusion, there was a statistically significant reduction in pain duration by 36 hours and a 3–5-fold reduction in analgesic use. 

 This was followed by a phase III RCT using poloxamer 188, with 255 individuals enrolled at 40 study sites [11]. In the poloxamer 188-treated group, there was a “modest” 9-hour reduction in pain duration, with more pronounced effects in children under 15 years old (21-hour reduction) and subjects receiving hydroxyurea (16-hour reduction). There were no significant differences between the poloxamer 188- versus placebo-treated groups in time to discharge, pain severity ratings, or total analgesic use. Pharmacokinetics showed mean steady state drug concentration “within the therapeutic range of rheological and antiadhesive effects.” The differences in responses between the phase II and III trials were attributed to the more stringent definition of pain duration used in the Phase III trial, and the authors concluded that poloxamer 188 may most benefit children with SCD under 15 years of age or those being treated with hydroxyurea. 

Close comparison shows differences between the Phase II and III trials. There was a shorter duration of pain prior to beginning study drug in the Phase II trial. The inclusion criteria for the Phase II trial included 4–18 hours of moderate pain on presentation, and there was a median of 17 hours between onset of pain and start of study infusion. In contrast, subjects in the phase III trial had 1.87–2.25 days of pain from onset of crisis to randomization, with an additional 2.3 hours between randomization to start of study infusion, or almost 3 times longer pain duration before study drug infusion was begun. In addition, the loading dose of poloxamer 188 was 3-fold higher in the phase II trial compared to phase III. Lastly, the phase II trial allowed nonsteroidal anti-inflammatory drugs (NSAIDs) for analgesia, but they were not allowed during the study drug infusion and for 12 hours following discontinuation in the phase III trial. The absence of opioid-sparing effect of NSAIDs may have contributed to the lack of difference in opioid usage.

### 2.2. Gardos Channel Inhibition

 Erythrocyte water content is an important determinant of sickle hemoglobin concentration and polymer formation. Erythrocyte hydration status is controlled by KCl and water loss through two transport systems, K-Cl cotransport and the calcium-dependent potassium, or Gardos channel. Gardos channel inhibition in sickle cells was found to improve erythrocyte hydration status [12]. The orally available antifungal agent, clotrimazole, could completely inhibit deoxygenation-induced Gardos channel-mediated K^+^ loss. Oral clotrimazole was found to reduce K^+^ loss and improve erythrocyte volume in transgenic mice expressing hemoglobin S, Antilles, and D Punjab (SAD mice) [13] and in a phase I-II trial in five adults with hemoglobin SS [14]. However, clotrimazole as a potential red cell hydrating agent was felt to be limited by its poor oral absorption, short half-life, and the development of elevated hepatic transaminases at high doses. 

 Based on the structure of clotrimazole, alternative agents were developed to more potently and specifically inhibit the erythrocyte Gardos channel. Senicapoc (ICA-17043) was found to inhibit SAD mouse red cell dehydration *in vitro* and *in vivo *and an increase in hematocrit of 7% [15]. A phase II RCT in humans showed favorable hematologic responses to senicapoc 10 mg daily compared to placebo, with 0.68 gm/dL increase in hemoglobin concentration and reduction in dense erythrocytes, reticulocytes, lactose dehydrogenase (LDH), and indirect bilirubin [16]. 

 This was followed by a phase III RCT with 297 adult subjects randomized at 75 study centers to receive senicapoc for 52 weeks [17]. The dose was 20 mg twice daily for four days, followed by 10 mg daily. The primary endpoint was the frequency of sickle cell-related painful crises requiring medical facility treatment. The study was discontinued early due to lack of efficacy. Despite improvement in hematologic parameters, there was no difference in painful crisis rates between subjects receiving senicapoc compared to placebo. The authors proposed that a possible reason for the discrepancy between hematologic responses and painful crisis rates was that reduction in hemolysis rate increases bioavailability of NO, which in turn enhances nocioceptive pain signaling. 

 Of note, the Gardos channel, also known as the intermediate conductance Ca^2+^-activated K^+^ channel, K_Ca_3.1, KCNN4, or SK4, is expressed in other cell types, such as T- and B-lymphocytes, macrophages, endothelial cells, fibroblasts, vascular smooth muscle cells, and neurons. In blood vessels, the K_Ca_3.1 channel plays a role in endothelium-derived hyperpolarizing factor- (EDHF-) induced vasodilation, which may play a major role in the microcirculation [18]. Chemical inhibition of the channel or KCNN4−/− knockout in mice resulted in hyperresponsiveness to stress due to enhanced adrenocorticotropic hormone (ACTH) secretion by the anterior pituitary [19]. The K_Ca_3.1 channel has approximately 50% homology with K_Ca_2 family channels, which are small conductance channels located in neurons and involved in afterhyperpolarization. These channels are expressed in high concentrations in the brain and are critical for learning and memory [20]. Therefore, it is possible that the unexpected results of the senicapoc trial may be related to the drug's effect on cell types other than sickle erythrocytes, particularly on EDHF-dependent vasodilation or in the nervous system. 

### 2.3. Inhaled Nitric Oxide for Acute Painful Episodes

Nitric oxide (NO) is an important vasoactive molecule, with effects on vascular smooth muscle dilation and modulating leukocyte and platelet activation. The use of nitric oxide in treatment sickle cell disease acute chest syndrome (ACS) was first reported in 1997 [21], followed by additional critical care medicine reports of clinical benefit in ACS, stroke, and multiorgan failure syndrome [22–24]. Cell-free hemoglobin has been found to reduce NO concentrations [25]. Individuals with hemoglobin SS had 20-fold higher plasma heme concentrations (4.2 *μ*M), and NO consumption was greater in sickle cell plasma and linearly correlated with plasma heme concentration. Forearm blood flow was lower in patients with hemoglobin SS, which improved after infusion of sodium nitroprusside (SNP), a NO donor. Giving inhaled NO at 80 ppm reduced NO consumption of sickle cell plasma by oxidizing and nitrosylating hemoglobin. Inhaled NO at 80 ppm was found to increase skin oxygenation in people with SCD but had no effect on forearm blood flow [26]. 

 In SAD mice, inhaled NO at 20 ppm improved survival rates during exposure to hypoxia when it was given 30 minutes prior to hypoxic exposure and continued during the entire exposure [27]. However, there was no benefit if a lower concentration of NO was used, or if it was given only before or only during hypoxia. The benefits of NO preinhalation and treatment during hypoxia-induced vaso-occlusion were proposed to include reduction of platelet and erythrocyte adhesion, but these mechanisms were not tested. 

 There have been three published studies of inhaled NO therapy for acute pain episodes in SCD. In the first study, 20 pediatric subjects with SCD were enrolled, 10 who were treated with inhaled NO 80 ppm for 4 hours and 10 with placebo [28]. The group who received inhaled NO had lower pain ratings and less morphine used at 6 hours, but no significant difference in duration of hospitalization. In the second study, nine adults with SCD were treated with inhaled NO 80 ppm for 4 hours and nine with placebo [29]. The group who received inhaled NO had lower pain ratings at six hours, but no difference in morphine utilization. A multicenter RCT of inhaled NO was conducted with 150 children and adults age 10 years and older with SCD presenting with acute pain crisis [30]. Inhaled NO was given for up to 72 hours, with an initial concentration of 80 ppm for the first four hours, 40 ppm for the next four hours, and then pulsed delivery at 5 ppm for the remainder of the 72 hour period. There was no difference between groups for the primary outcome measure, time to resolution of pain, or duration of hospitalization, pain scores at 24 hours, or total opioid use. 

## 3. Lost in Translation?

 With the strength of the preclinical findings, why were these therapeutic strategies not effective in reducing the impact of acute pain, the most common complication of sickle cell disease? Sickle cell clinical research probably suffers from the same challenges as other clinical trials in the United States. In 2012, the Institute of Medicine published a report that discusses the major challenges with current clinical trials, including high costs due to elaborate administrative procedures, failure to enroll sufficient numbers of participants, regulatory issues, and failure to publish negative results [31]. For sickle cell disease research, it has been recommended that clinical trials design be improved, especially to ensure sufficient enrollment, redefine study endpoints, and account for different clinical subphenotypes [4]. 

 In the studies reviewed in this paper, the outcome measures for acute pain were different in many of the studies conducted. The need to use multiple study sites to accommodate the number of subjects necessary for large clinical trials makes consistency of study treatments and outcome measures extremely important. For example, in the phase III inhaled NO RCT, there were institutional differences between study sites, such that two sites had significantly different outcomes than the others. In the poloxamer 188 studies, there were several methodologic differences between the phase II and III trials which may account for the disparity in efficacy results.

### 3.1. Lack of Models for Acute Pain in Sickle Cell Disease

Beyond challenges inherent to clinical trials, there are some recurring themes in these studies. First, preclinical studies were surrogates, but not sufficiently good model systems, for actual pain episodes. In general, preclinical *in vivo* evidence of the relevance of individual pathways on important sickle cell outcomes has been lacking. For antiadhesion therapy with poloxamer 188, the general “masking” effect was a good approach to address the redundancy of multiple red cell, leukocyte, platelet, and endothelial cell adhesion molecules, but the nearly complete endothelial adhesion blockade demonstrated *in vitro* had not been reported in published animal studies prior to human clinical trials. In the case of Gardos channel inihibition, there was strong evidence for improvement in red cell volume, reduced hemolysis, and anemia, but no testing in a model mimicking painful crisis prior to the phase III clinical trial. Preclinical data for inhaled NO showed beneficial effects on survival in hypoxic conditions in transgenic sickle cell mice, but it was only helpful when given before hypoxia was initiated. Inhaled NO had no effect on forearm blood flow, but somewhat improved skin hemoglobin oxygen saturation. In healthy adults, it has been shown that inhaled NO 40 ppm corrects hypoxia-induced pulmonary hypertension without an effect on systemic vasodilation [32]. While it would be predicted that inhaled NO would promote vasodilation, reduce inflammation, and improve red cell hydration, none of these outcomes were reported in the three inhaled NO clinical trials, making it difficult to ascertain whether there was any improvement to these proposed mechanisms resulting from treatment. 

 At the heart of the matter, it remains an article of faith that sickle cell polymerization and microvascular occlusion is the actual cause of acute pain in SCD. There is currently no way to visualize vaso-occlusion to corroborate that sites of sickle erythrocyte microvascular occlusion correspond to locations where patients feel pain or to show that therapies that restore or maintain normal blood flow will relieve vaso-occlusion and pain. Intravital microscopy of various vascular beds in rodents has been used to study agents that block adhesion of sickle erythrocytes and leukocytes, including *ex vivo* rat mesenteric vessels, transgenic mouse cremaster, and brain window [33–35]. While vascular occlusions can be visualized, animals are anesthetized during the procedures and cannot be evaluated for pain. In humans, conjunctival and skin blood flow have been shown to be abnormal in people with SCD [26, 36], but these are not typically sites of pain, and blood flow has not been specifically measured at these sites during acute pain episodes. Bulbar conjunctival blood flow was found to improve after erythrocytapheresis [37] and in a small number of subjects treated with poloxamer 188 [38]. 

### 3.2. Drug Delivery and Timing

 Even if a therapy is effective in the person with SCD, there may be difficulty in maintaining effective pharmacokinetics, timing of therapy, and/or drug delivery to the site of vaso-occlusion. A treatment may work well at certain concentrations *in vitro* or in transgenic mice, but these same concentrations may not be achievable in the setting of a person with a larger volume of distribution for the necessary duration of the acute painful crisis. In early nitric oxide inhalation studies, acute treatment with inhaled NO 80 ppm was for 4 hours, and followup was for 4–24 hours. It is possible that these time courses were too short to be effective, since vaso-occlusion commonly does not resolve in 6 hours. In the case of established tissue injury from ischemia, pain may not necessarily resolve within a day. In the larger randomized controlled trial of inhaled NO, the equivalent of NO 5 ppm was given for up to 72 hours, an overall lower concentration, not previously reported to have an effect on vasodilation or inflammatory markers, and the authors state that this lower rate of administration may have been insufficient to generate systemic nitrite, an NO metabolite which may mediate its tissue effects. 

As in stroke therapy with neuroprotectants and thrombolytics [39], timing of therapy in acute painful crisis may be an important factor in effectiveness. Sickle cell pain may be similar to myocardial and cerebral infarction, in that the benefits of therapies to reverse vessel occlusion and restore perfusion need to be initiated very early in the ischemic process to be effective, and otherwise the ischemic injury is established and cannot be readily reversed. We currently have no way of knowing whether there is still ongoing vaso-occlusion when a person with SCD presents for treatment of pain, if the pain is instead associated with reperfusion or due to persistent transmission from afferent sensory neurons that have been activated in ischemic tissues. If pain is a postocclusive event, then treatments that reduce sickle cell polymerization, adhesion, thrombosis, or vaso-constriction may not have much effect on the acute pain experience, though they may prevent worsening or progressive vaso-occlusion.

 In the hemoglobin SAD mouse hypoxia studies, inhaled NO was only effective in preventing mortality when the animals were pretreated for 30 minutes prior to hypoxic exposure, which would not be feasible in people. Nitric oxide may be effective if therapy is begun at the very outset of vaso-occlusion *and* if vaso-constriction and inflammation are important inciting factors. Individuals in the clinical trials may present for treatment to the clinical research site several days after the initiation of the vaso-occlusive event, after unsuccessful home therapy. A similar effect of timing was seen in poloxamer 188 studies, where the drug was more effective in the Phase II trial when it was given earlier in the onset of the painful episode.

### 3.3. Complexity of Sickle Cell Disease

 Lastly, it is likely that therapies which target specific components of sickle cell pathophysiology do not sufficiently inhibit the entire process that makes up acute pain episodes. Poloxamer 188 has the potential to inhibit multiple cell-cell interactions, but adhesion has not yet been demonstrated to be a major mechanism contributing to acute painful episodes. While the effects of senicapoc on sickle erythrocyte hydration and total hemoglobin concentration has been consistent between *in vitro* experiments, transgenic mice, and humans, it may also have effects on the K_Ca_3.1 channels of vascular cells and/or neurons that could adversely affect acute painful episodes. Inhaled NO could potentially improve blood flow by vasodilating vessels and reduce inflammation and platelet adhesion, but it may also promote cyclic GMP-mediated nociceptive pain transmission. Even if each of these therapies had fully beneficial effects on vaso-occlusion, alone they may not be adequate to reverse a painful crisis in progress if vessels are completely occluded with irreversibly sickled erythrocytes or if the pain is due to reperfusion response rather than vaso-occlusion. 

 Early reports discussing complexity in SCD date back to 1974 and 1983 [40, 41]. Frenette in 2002 described sickle cell vaso-occlusion is as a “multistep and multicellular paradigm,” involving sickle erythrocyte and leukocyte adherence in addition to sickle hemoglobin polymerization [42]. In 2009, HebbeI provided a detailed review of the many “subbiologies” that are involved in sickle cell vaso-occlusion and recommended multimodality chemoprophylaxis to target them simultaneously [43], a recommendation reiterated by others [4, 44]. Complexity in SCD has been recognized for several decades, but for practical considerations and the mandate for feasible hypothesis-driven research, most SCD research has focused on understanding and intervening in individual pathways. 

 To date, systems biology high-throughput and large dataset methodologies, or “omics” studies, of SCD have included transcriptome analysis of blood outgrowth endothelial cells, monocytes and reticulocytes [45] in humans, and kidneys in transgenic sickle cell mice [46]. Blood outgrowth endothelial cell transcriptome analysis showed that individuals with SCD and arterial occlusion in the Circle of Willis had higher expression of genes regulated by NFK-B and RelA, regulators of inflammation [47]. Sickle cell monocytes demonstrated differential expression of genes involved in heme metabolism, cell-cycle regulation, antioxidant and stress responses, inflammation, and angiogenesis [48]. Proteome analysis of sickle cell erythrocytes and plasma have shown upregulation of antioxidant proteins, an increase in cytoskeletal defects, an increase in protein repair and turnover components, a decrease in lipid raft proteins, and apolipoprotein dysregulation [49]. Genome-wide association studies (GWAS) were performed using DNA from over 1000 subjects in the Comprehensive Study of Sickle Cell Disease cohort [50]. Several genes that were identified were associated with severity of SCD symptoms and certain complications. Epigenetic analysis of reticulocytes before and after hydroxyurea demonstrated increased expression of microRNAs (miR)-26b and miR-151-3p to be associated with fetal hemoglobin response [51].

## 4. A Systems and Network Approach to Sickle Cell Disease

 Reductionism in science dates back to René Descartes in the 1600s [52]. His approach was to “divide all the difficulties under examination into as many parts as possible… beginning with simplest and most easily understood objects, and gradually ascending… to the knowledge of the most complex.”

 Systems theory was first coined by Ludwig von Bertalanffy in the 1940s, defining it as the transdisciplinary study of the abstract organization of phenomena, independent of their substance, type, or spatial or temporal scale of existence. It investigates both the principles common to all complex entities and the (usually mathematical) models which can be used to describe them [53].

 Donella Meadows defines a system as “a set of things interconnected in such a way that they produce their own pattern of behavior over time. The system may be buffeted, constricted, triggered, or driven by outside forces. But the system's response to these forces is characteristic of itself, and that response is seldom simple in the real world… Our own bodies are magnificent examples of integrated, interconnected, self-maintaining complexity [54].” Key definitions used in systems models include *stocks*, which are measurable materials or information; *flows*, or the movement of stocks, including inflow and outflow; and *feedback loops*, closed chains of causal connections from a stock that regulate the behavior of stocks. The concept of stabilizing or balancing feedback loops is analogous to its physiologic definition, where balancing feedback is required for homeostasis. In sickle cell disease, an example would be the interaction between free heme released by hemolysis and natural heme scavengers, such as haptoglobin and hemopexin. Health reflects a state of balanced equilibrium, whereas illness results from disequilibrium or loss of homeostasis. 

 Systems biology is the application of systems thinking to the study of biologic processes. Kitano wrote that systems biology is a ““holistic” approach to interconnect different cellular processes, such as metabolism and genetic regulation, instead of traditional reductionist methods [55].” Breitling states that it is “based on the comprehensive study of the molecular diversity of living systems, both natural and synthetic, the identification of simplifying general principles and patterns that are recurring features in living and engineered systems, and the integration of our biological knowledge in complex models of the regulatory networks that characterize life [56].” Machado defined that “systems biology is used to model complex biological processes using computational tools and high throughput experimental data [57].” However, Joyner warns that systems biology methods need to be used in the context of physiologic principles, such as homeostasis, feedback, redundancy, and adaptation, rather than applied generically and without guiding hypotheses [58].

 Network thinking was defined by Mitchell as “focusing on relationships between entities rather than the entities themselves [52].” A network is a collection of *nodes* connected by *links. *Nodes to connected to many others are *hubs. *Hubs are highly vulnerable to failure or can be targeted for attack. Examples of nodes are cells or molecules, and links can be relationships or physical connections, such as synapses, fibers, or routes. Alon has applied engineering principles to model biologic functions [59]. He wrote that, “Simplicity occurs in biologic networks. There are only a few types of recurring interactions, or *network motifs*. Each motif can perform defined information processing functions… Most biologic functions are carried out by specific groups of genes and proteins which form *functional modules*. For example, proteins work in coregulated groups such as pathways and complexes. This is analogous to modules in engineering, subroutines in software, or replaceable parts in machines. The working definition of a module *is a set of nodes that have strong interactions and a common function*.” 

**Figure 1:**
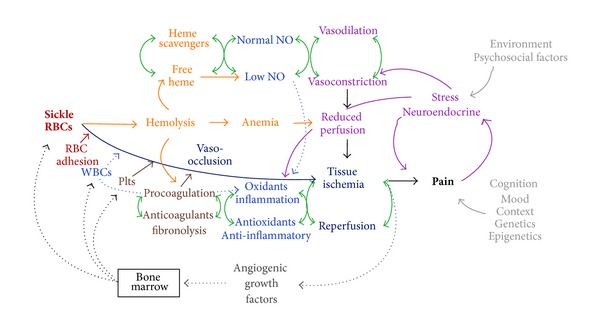
Acute pain model. This diagram shows proposed interactions between pathophysiologic mechanisms in sickle cell disease that lead to acute painful episodes. Key to mechanisms: green: balancing feedback loops, red: erythrocytes, orange: hemolysis, light blue: inflammation and oxidant stress, dark blue: ischemia and reperfusion, lavender: vasomotor, brown: coagulation, gray: angiogenesis, and light gray: pain modifiers. See text for details.

**Figure 2:**
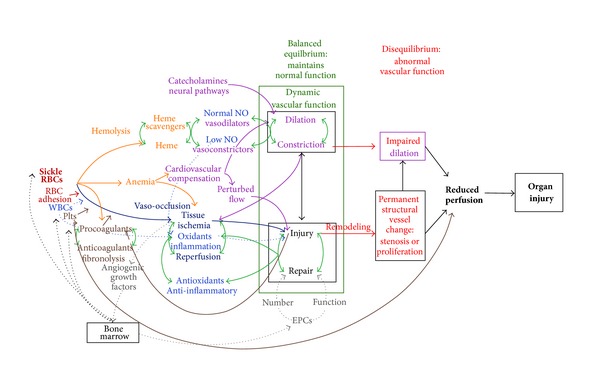
Chronic vasculopathy model. This diagram shows proposed interactions between pathophysiologic mechanisms in sickle cell disease that lead to chronic vasculopathy and organ injury. Key to mechanisms: Green arrows: balancing feedback loops, Red: abnormal vascular structure and function, orange: hemolysis, light blue: inflammation and oxidant stress, dark blue: ischemia and reperfusion, lavender: vasomotor, brown: coagulation, and gray: angiogenesis. See text for details.

 These concepts of systems thinking and network analysis were used to develop diagrammatic models that incorporate and which show potential interactions between multiple mechanisms putatively involved in acute pain episodes (Figure 1) and chronic vasculopathy (Figure 2) in SCD. These are not classic biologic network models with specific proteins or genes as nodes but include as modules major mechanistic pathways in SCD for which there is existing evidence. In these models, the primary defect of sickle hemoglobin polymerization causes secondary processes of hemolysis and vaso-occlusion, which further transduce their effects to blood vessels and organs through oxidative, inflammatory, vasomotor, coagulation, and angiogenic intermediaries. The roles of balancing feedback loops in homeostasis and the effects on the vascular bed are central features of the models. 

### 4.1. Acute Pain Model

Figure 1 shows potential relationships between mechanistic pathways in SCD that lead to acute pain episodes. The most commonly considered pathway in this process begins with sickle hemoglobin polymerization, leading to formation of rigid sickle erythrocytes and microvascular occlusion when these cells become trapped in small vessels. In the presence of inflammation (infection or ischemia and reperfusion), vascular endothelial cells express a number of adhesion molecules that facilitate adherence of mature erythrocytes, reticulocytes, activated leukocytes, and/or platelets. Endothelial tissue factor expression can activate coagulation factors and thrombosis. 

 Hemolysis can also contribute to vaso-occlusion, through the release of free heme, reactive iron species, and membrane microparticles. Free heme can bind NO and reduce its bioavailability, which promotes vaso-constriction, inflammation, and platelet aggregation. Heme and reactive iron species can directly cause injury and oxidative damage to endothelium. Erythrocyte membrane microparticles with exposed phosphatidylserine may active platelets and promote coagulation. Hemolytic anemia reduces oxygen delivery to tissues and contributes to reduced tissue and organ perfusion chronically, likely leaving them vulnerable to the effects of acute vaso-occlusion.

 After vaso-occlusion has occurred, there is local tissue ischemia and reperfusion, which includes inflammatory and oxidant responses. Our group has demonstrated elevated levels of hypoxia-inducible factor- (HIF-) associated angiogenic growth factors in children with hemoglobin SS during steady state [60], suggesting a chronic baseline state of tissue ischemia even in the absence of symptomatic pain or acute complications. In particular, levels of stromal-derived factor- (SDF-) 1*α*, produced by ischemic endothelium and other cells, were associated with the number of bone marrow-derived circulating progenitors with angiogenic potential (CD34^+^/VEGFR2^+^) and total white blood cell (WBC) count. Total WBC has been found to be associated with SCD severity in some series, and the relationship between WBC and SDF-1*α* suggests that those people with the most ongoing tissue ischemia may have the most vaso-occlusive organ injury. Tissue ischemia has a reinforcing effect on inflammation, through HIF-mediated angiogenic stimulation of WBC release from the bone marrow. 

 Pain is itself a complex process and is not diagrammed in detail in this model. The pain experience is the product of the nociceptive input from injured tissue but also involves cognitive, contextual, mood, and individual differences, such as sex, age, and genetics. Stress related to acute pain can induce neuro-endocrine responses, such as stress steroids, catecholamines, and pain peptides (substance P, neurokinins). Catecholamines promote vaso-constriction, and the pain peptides can be proinflammatory. The current model also does not attempt to delineate all of the external factors that may also influence a pain episode, such as environment (ambient temperature, second-hand smoke) or psychosocial factors. Neuropathic pain resulting from ischemic injury directly to nerves may account for some of the pain experienced in SCD and would not necessarily respond to antisickling therapies or those targeting inhibition of vaso-occlusion. Chronic pain may involve “imprinting” of the nervous system by epigenetic modifications (DNA methylation, histone modification) that regulate gene expression [61]. Chronic pain and opioids can alter the structure of the brain [62, 63]. To summarize, there are many pathways that can interact in acute pain episodes in SCD, some positively reinforce others, and some factors are outside of the body.

### 4.2. Chronic Vasculopathy Model

 A model with similar features describes the development of chronic vasculopathy in SCD (Figure 2). In the vasculopathy model, dynamic vascular function is the central process affected by SCD derangements. In the healthy state, there is balanced equilibrium between vasodilation and constriction, and between endothelial injury and repair. 

 Hemolysis will cause disequilibrium in favor of vasoconstriction, and longterm exposure can eventually cause sustained impairment in vasodilation and reduced vessel compliance (stiffness). Early stage remodeling may cause arterial wall stiffness and reduced compliance before arterial stenosis is apparent in imaging studies. This may be the case in those children with elevated transcranial doppler (TCD) velocities in the cerebral arteries that is associated with high stroke risk, but whose brain magnetic resonance arteriography (MRA) shows no arterial stenoses. 

 Anemia reduces oxygen delivery and organ perfusion. Ischemia and reperfusion due to the combination of anemia and repetitive vaso-occlusion stimulate HIF-associated angiogenic growth factors. Anemia-associated compensatory circulatory changes are likely to cause disturbed blood flow, which can promote endothelial injury, and potentially contribute to vessel wall remodeling (intimal proliferation and arterial stenosis). Our group has demonstrated elevated serum concentrations of platelet-derived growth factor- (PDGF-) AA, a mediator of vascular remodeling, and brain derived neurotrophic factor (BDNF), a biomarker of cerebral ischemia, in children with hemoglobin SS and high TCD velocities [64], consistent with the model. High frequency of red cell transfusion therapy reduced PDGF-AA, soluble VCAM-1, and RANTES in children with hemoglobin SS and high TCD velocities, suggesting that correction of anemia and reduction in sickle cells by transfusion reduces vascular injury, inflammation, and vascular remodeling responses [65]. 

Considering sickle cell disease through the lenses of these models helps explain the potential limitations of therapies that are targeted to single pathways. While the therapies discussed earlier may have some multimodal effects, there are many additional contributors to acute pain that were not modulated by individual therapy. These models contrast conceptually to the bimodal model proposed by Kato, in which certain SCD complications are primarily related to either hemolysis or vaso-occlusion, and that individuals exhibit one or the other subphenotype [66]. In these models, hemolysis and vaso-occlusion occur simultaneously in both acute pain and chronic vasculopathy, although not necessarily equally in magnitude at any given time. 

 Complexity in SCD does not necessarily mean that the disease is hopelessly complicated and cannot be successfully treated. In these models, sickle hemoglobin polymerization is the network hub, and therefore most vulnerable to attack (correction). This is consistent with the clinical observation that red cell transfusions and hydroxyurea, which correct hemoglobin S polymerization and/or sickled erythrocytes, are often effective SCD treatments and also reduce downstream mediators. However, while hydroxyurea is an alternative to transfusions for certain SCD indications, it is not an equivalent therapy. In the setting of established structural vascular disease, such as significant cerebral vasculopathy in individuals with stroke, hydroxyurea therapy in combination with phlebotomy to relieve iron overload was not as effective as transfusions in preventing stroke [67]. Even chronic red blood cell transfusions are not effective in preventing recurrent stroke in up to 20% of individuals [68], suggesting additional unidentified stroke mediators in a subset of individuals with the most severe disease. 

 Viewed from this perspective, replacement of sickle erythrocytes by stem cell transplantation, gene therapy correction of the hemoglobin S mutation, or very effective fetal hemoglobin induction are likely to be the most effective SCD treatments in the long run. However, until these are widely available, should severe sickle cell disease be treated like thalassemia, with lifelong chronic red cell transfusions? How do we identify those at highest risk who would benefit from life-long transfusions begun early in life?

## 5. Future Directions

 This paper of selected clinical trials and discussion of complexity in SCD has identified some challenges in the search for alternative effective therapies. There are logistical and methodological issues related to clinical trials, such as consistent study endpoint definition and effective timing and delivery of therapeutics; lack of good model systems in which to test the effect of therapeutics; and the larger problem posed by complexity—it is difficult to shut down all aspects of the system at once without using transfusions or stem cell transplantation. Acknowledgment of complexity does not imply that therapies targeting individual intermediary mechanisms in SCD should be abandoned but necessitates that their effectiveness needs to be tested in highly predictive model systems prior to embarking on large-scale clinical trials. In this section, some possible approaches to these challenges are suggested.

### 5.1. Strategic Mechanistic Testing of Therapeutics in the Transgenic Sickle Cell Mouse Model

 There is a need to develop preclinical model systems for complications such as acute pain or chronic vasculopathy that are highly predictive of those processes in people with SCD. There are currently transgenic sickle cell mouse models of acute chest syndrome and pulmonary hypertension, but none yet for SCD-associated stroke or acute painful crisis. The most extreme model used in transgenic sickle cell mice is hypoxia-induced death, which presumably occurs from sickle cell-induced vaso-occlusion in the entire animal. While not similar to human acute pain episodes in severity, drugs that are potent enough to prevent this degree of sickling or vaso-occlusion should be effective in less extreme situations. For example, the lack of beneficial effect of inhaled NO in the phase III RCT was accurately predicted by the lack of rescue in the hypoxia-exposed SAD mice who were treated with only posthypoxia inhaled NO. If there was a way to image sites of pain while simultaneously measuring pain behaviors in the sickle cell mouse, it might be useful to titrate the severity of the hypoxic exposure to a sublethal dose that might simulate acute painful events.

 Transplantation of sickle cell mouse bone marrow or injection of sickle mouse erythrocytes into a transgenic strain with a desired gene knockout may be used to prove the essential role of an individual molecule in sickle cell pathogenesis. These approaches have been used to demonstrate the role of P-selectin in sickle cell microvascular occlusion [69] and superoxide produced by NADPH oxidase in cerebral artery microvascular occlusions [35]. Tissue-specific conditional knockouts in a transgenic sickle cell mouse would be an elegant but technically challenging experimental approach to test the role of individual molecules in the “native” environment of the transgenic sickle cell mouse. However, such an approach should only be used in pathways in which there is no physiologic redundancy. At least ten different sickle erythrocyte membrane proteins or components have been identified as potentially involved in adhesion or interaction with endothelial cells [70], so that knocking down only one is unlikely to have major impact on vaso-occlusion, unless it is a *critically* important molecule that has greater impact than all of the others in combination. 

### 5.2. Develop Imaging Systems for Vaso-Occlusion

 One of the most basic tenets of the field is that acute pain episodes are caused by the occlusion of microvessels by sickled erythrocytes. While this makes theoretic sense on the basis of hemoglobin polymerization, rheologic and microvascular studies, this has never been proven in people with SCD. When does acute pain begin relative to vaso-occlusion? Do individuals with full body pain really have sickling everywhere? Does vaso-occlusion in one part of the body induce pain at distant sites through crosstalk between neurons in the sensory pathway or central nervous system? The field needs ways to visualize both sites of vaso-occlusion and pain pathways to demonstrate that they are actually related and to have an outcome measure for testing pain treatments. Vaso-occlusive sites could potentially be visualized by radionuclide-tagged particles that home to ischemic tissue markers, such as SDF-1*α*. 

### 5.3. Interdisciplinary Pain Research

The pain research field apparently has similar challenges in developing chronic pain treatments as has been described here for SCD. Borsook wrote “Drug development for pain often fails, paralleling many other CNS areas, because preclinical and experimental clinical proof-of-concept (POC) studies do not translate well to clinical conditions and patient populations [71].” Proposed biomarkers for pain include functional brain imaging with analysis of focal brain regions and chemical biomarkers, such as CNS neurotransmitters (e.g., glutamate, GABA, and glycine) and brain metabolites (e.g., NAA, choline), which can be measured *in vivo* using magnetic resonance spectroscopy (MRS) [72]. Functional MRI (fMRI) with support vector machine learning analysis of the whole brain is able distinguish pain without the need for verbal communication [73]. Since pain is such a large part of SCD symptomatology, future research collaborations with established pain neurobiologists equipped to use these state-of-art approaches are warranted as suggested by the NIH Blueprint for Neuroscience “Grand Challenge on Pain [74].” 

### 5.4. Develop a Consensus Set of Meaningful Study Endpoints and Test for Mechanistic Markers during Clinical Trials

 For agents that prevent pain, the number and duration of acute painful episodes, including both facility- and home-treated events, and quality of life measures related to pain may be a more accurate assessment of the true effectiveness of a pain prevention therapy. In the treatment of acute pain, there needs to be agreement on the definition of who should be hospitalized for therapy to assure comparability of groups and on the definition of what constitutes “resolution of pain.” It has also been recommended that study endpoints better match the mechanism of action of the therapeutic [4, 44]. For example, pain outcomes may not be applicable to agents that reduce hemolysis (Gardos channel inhibitors) or target NO signaling, since they may be more likely to be beneficial in the vasculopathy subphenotype (pulmonary hypertension, priapism). Analogously, there needs to be consensus on vasculopathy endpoints. For example, is echocardiographic measurement of tricuspid regurgitant velocity acceptable as a noninvasive surrogate of pulmonary hypertension? In the case of any endpoint, does it respond rapidly enough to therapy to be feasibly measured as a study outcome?

 To better understand the role of therapeutic mechanisms, biomarkers or other functional outcome measures should be included as part of early phase clinical trials. As mentioned in the earlier discussion of inhaled NO, none of the published clinical trials reported on vasodilation, platelet aggregation, or inflammatory biomarkers, such as sVCAM. Senicapoc's red cell effects were consistently measured in each clinical trial, so that lack of efficacy in reducing pain frequency appears to be unrelated to improvement in cell hydration status. In this situation, the data helps guide investigators in the analysis of therapeutic and unwanted side effects. Measurement of biomarkers makes clinical trials more labor intensive and is unlikely to be feasible at every study site. However, it provides important evidence to explain drug efficacy or lack thereof. 

### 5.5. Directed Delivery of Therapeutics to Ischemic Sites

 In combination with biomarker and functional assays, early phase clinical trials should include well-described pharmacokinetics to establish that the administered doses result in plasma concentrations that are comparable to those used in preclinical studies. However, systemic concentrations of a drug may not be the same as the amount delivered to the affected tissues, especially in areas of reduced perfusion. There is an opportunity to apply nanotechnology to selectively deliver analgesics, antisickling, other vaso-occlusion disrupting agents, or drugs that improve tissue oxygenation to ischemic regions (presumably corresponding to vaso-occlusion) by targeting ischemia markers, such as SDF-1*α*. 

### 5.6. Systems Biology, “Omics,” and Computational Modeling

 Systems biology and “omics” technologies may be useful in understanding complex biologic systems. This approach is in its infancy in SCD and could be applied to dissecting specific problems, such as identifying gene variants or microRNAs that predispose to higher risk of well-defined complications such as stroke, chronic pain, changes in gene expression, or epigenetic modifications that occur with therapies. Another goal for the application of “omics” to SCD would be to identify master regulators that control multiple pathophysiologic mechanisms, so that these could be targeted for inhibition. 

 Predictive computational models can potentially be used to integrate multiple types of patient data, such as laboratory values, radiographic findings, circulating biomarkers, and genetic and epigenetic data, with disease phenotype to define risk categories. The ability to identify individuals at highest risk for severe complications would allow the option of early treatment with high-risk therapies, currently red blood cell transfusion or stem cell transplantation, well before the onset of complications. The predictive strength of such modeling approaches would be enhanced by including as many individuals with SCD as possible, perhaps through a collaborative national data registry and biorepository system.

### 5.7. Improve Understanding of the Effects of Psychosocial Determinants and the Environment on SCD Complications

It has been well described that stresses related to poverty and racism affect cardiovascular risk and disease and disproportionately affect African Americans. It is very likely that such gene-environment interactions are additional factors influencing SCD complexity and outcomes and are currently not adequately understood. For example, how do chronic undernutrition, lack of utilities, poverty, or personal or familial mental illness affect the frequency and severity of acute illness or the response to therapy in a person with SCD? Epidemiologic methodologies including geocoding could be applied to studying some of these factors in SCD. Such variables can be added to computational predictive models to help us begin to understand the relative contributions of factors in SCD complications.

## 6. Conclusions

SCD is caused by a single mutation in beta globin but triggers several pathophysiologic pathways and results in a highly complex disease. This complexity is likely to be one of the major barriers to the development of successful new treatments which, to date, has largely concentrated on individual mechanistic pathways. Future development of therapeutics needs to continue to focus on correcting the underlying problem of sickle hemoglobin polymerization but should also include development of better model systems for acute and chronic SCD complications, methods for visualizing and measuring vaso-occlusion and associated pain, directed delivery of therapies to sites of vaso-occlusion, systems biology approaches to identify master regulators of the multiple downstream effectors of hemolysis and vaso-occlusion, and better understanding of the contribution of gene-environment interactions on sickle cell disease complications. Considering the number of pathophysiologic processes caused by SCD, it is astonishing how well the body maintains homeostasis sufficient for growth, development, and general health for periods between acute illnesses. The approach to this disease should also include an effort to identify mechanisms that are crucial to maintaining homeostasis and wellness. While there have been many life-saving advances in the treatment of SCD, much work remains to achieve the goal of curing the disease and developing safe and effective therapies to improve health and well-being.

## Figures and Tables

**Table 1:** Major types of sickle cell intervention studies registered on the website http://www.clinicaltrials.gov/, as of December 1, 2012, of a total of 96 trials.

Pathway/mechanism	Number of studies
Bone marrow transplantation	21
Hemoglobin F induction	9
Nitric oxide related	8
Analgesic regimens	6
Nutritional supplements	4
Adhesion inhibition	3
Transfusion therapy	3
Red cell hydration	3
Noninvasive ventilation	3
Statins	2
Renin-angiotensin pathway in nephropathy	2
Iron chelation	2
Educational tools	2
Antiinflammation	2
Gene transfer	1
Carbon monoxide donation	1
Anti-coagulation	1

## References

[1] Sickle Cell Disease—Data and Statistics. http://www.cdc.gov/ncbddd/sicklecell/data.html.

[2] Platt OS, Brambilla DJ, Rosse WF (1994). Mortality in sickle cell disease—life expectancy and risk factors for early death. *The New England Journal of Medicine*.

[3] (2002). *Sickle Cell Research for Treatment and Cure*.

[4] Vichinsky E (2012). Emerging “A” therapies in hemoglobinopathies: agonists, antagonists, antioxidants, and arginine. *Hematology American Society of Hematology Education Program*.

[5] Hoots WK, Shurin SB (2012). Future directions of sickle cell disease research: the NIH perspective. *Pediatric Blood and Cancer*.

[6] Hankins J, Aygun B (2009). Pharmacotherapy in sickle cell disease—state of the art and future prospects. *The British Journal of Haematology*.

[7] Raphael RI (2005). Pathophysiology and treatment of sickle cell disease. *Clinical Advances in Hematology and Oncology*.

[8] Padilla F, Wear JO, van Wagner WH (1975). Effect of fluorocarbon emulsions on the mechanical fragility of normal and sickle cells: in vitro studies. *Federation Proceedings*.

[9] Smith CM, Hebbel RP, Tukey DP, Clawson CC, White JG, Vercellotti GM (1987). Pluronic F-68 reduces the endothelial adherence and improves the rheology of liganded sickle erythrocytes. *Blood*.

[10] Adams-Graves P, Kedar A, Koshy M (1997). RheothRx (Poloxamer 188) injection for the acute painful episode of sickle cell disease: a pilot study. *Blood*.

[11] Orringer EP, Casella JF, Ataga KI (2001). Purified poloxamer 188 for treatment of acute vaso-occlusive crisis of sickle cell disease: a randomized controlled trial. *The Journal of the American Medical Association*.

[12] Brugnara C, de Franceschi L, Alper SL (1993). Inhibition of Ca^2+^-dependent K^+^ transport and cell dehydration in sickle erythrocytes by clotrimazole and other imidazole derivatives. *Journal of Clinical Investigation*.

[13] de Franceschi L, Saadane N, Trudel M, Alper SL, Brugnara C, Beuzard Y (1994). Treatment with oral clotrimazole blocks Ca^2+^-activated K^+^ transport and reverses erythrocyte dehydration in transgenic SAD mice. A model for therapy of sickle cell disease. *Journal of Clinical Investigation*.

[14] Brugnara C, Gee B, Armsby CC (1996). Therapy with oral clotrimazole induces inhibition of the Gardos channel and reduction of erythrocyte dehydration in patients with sickle cell disease. *Journal of Clinical Investigation*.

[15] Stocker JW, de Franceschi L, McNaughton-Smith GA, Corrocher R, Beuzard Y, Brugnara C (2003). ICA-17043, a novel Gardos channel blocker, prevents sickled red blood cell dehydration in vitro and in vivo in SAD mice. *Blood*.

[16] Ataga KI, Smith WR, de Castro LM (2008). Efficacy and safety of the Gardos channel blocker, senicapoc (ICA-17043), in patients with sickle cell anemia. *Blood*.

[17] Ataga KI, Reid M, Ballas SK (2011). Improvements in haemolysis and indicators of erythrocyte survival do not correlate with acute vaso-occlusive crises in patients with sickle cell disease: a phase III randomized, placebo-controlled, double-blind study of the Gardos channel blocker senicapoc (ICA-17043). *The British Journal of Haematology*.

[18] Tharp DL, Bowles DK (2009). The intermediate-conductance Ca^2+^-activated K^+^ channel (KCa3.1) in vascular disease. *Cardiovascular and Hematological Agents in Medicinal Chemistry*.

[19] Liang Z, Chen L, McClafferty H (2011). Control of hypothalamic-pituitary-adrenal stress axis activity by the intermediate conductance calcium-activated potassium channel, SK4. *Journal of Physiology*.

[20] Kuiper EF, Nelemans A, Luiten P, Nijholt I, Dolga A, Eisel U (2012). K(Ca)2 and k(ca)3 channels in learning and memory processes, and neurodegeneration. *Frontiers in Pharmacology*.

[21] Atz AM, Wessel DL (1997). Inhaled nitric oxide in sickle cell disease with acute chest syndrome. *Anesthesiology*.

[22] Sullivan KJ, Goodwin SR, Evangelist J, Moore RD, Mehta P (1999). Nitric oxide successfully used to treat acute chest syndrome of sickle cell disease in a young adolescent. *Critical Care Medicine*.

[23] Montero-Huerta P, Hess DR, Head CA (2006). Inhaled nitric oxide for treatment of sickle cell stroke. *Anesthesiology*.

[24] Chang WL, Corate LM, Sinclair JM, van der Heyde HC (2008). Continuous inhaled nitric oxide therapy in a case of sickle cell disease with multiorgan involvement. *Journal of Investigative Medicine*.

[25] Reiter CD, Wang X, Tanus-Santos JE (2002). Cell-free hemoglobin limits nitric oxide bioavailability in sickle-cell disease. *Nature Medicine*.

[26] Zuzak KJ, Gladwin MT, Cannon RO, Levin IW (2003). Imaging hemoglobin oxygen saturation in sickle cell disease patients using noninvasive visible reflectance hyperspectral techniques: effects of nitric oxide. *The American Journal of Physiology*.

[27] Martinez-Ruiz R, Montero-Huerta P, Hromi J, Head CA (2001). Inhaled nitric oxide improves survival rates during hypoxia in a sickle cell (SAD) mouse model. *Anesthesiology*.

[28] Weiner DL, Hibberd PL, Betit P, Cooper AB, Botelho CA, Brugnara C (2003). Preliminary assessment of inhaled nitric oxide for acute vaso-occlusive crisis in pediatric patients with sickle cell disease. *The Journal of the American Medical Association*.

[29] Head CA, Swerdlow P, McDade WA (2010). Beneficial effects of nitric oxide breathing in adult patients with sickle cell crisis. *The American Journal of Hematology*.

[30] Gladwin MT, Kato GJ, Weiner D (2011). Nitric oxide for inhalation in the acute treatment of sickle cell pain crisis: a randomized controlled trial. *The Journal of the American Medical Association*.

[31] Institute of Medicine (2012). *Envisioning a Transformed Clinical Trials Enterprise in the United States: Establishing an Agenda for 2020*.

[32] Frostell CG, Blomqvist H, Hedenstierna G, Lundberg J, Zapol WM (1993). Inhaled nitric oxide selectively reverses human hypoxic pulmonary vasoconstriction without causing systemic vasodilation. *Anesthesiology*.

[33] Chang J, Patton JT, Sarkar A, Ernst B, Magnani JL, Frenette PS (2010). GMI-1070, a novel pan-selectin antagonist, reverses acute vascular occlusions in sickle cell mice. *Blood*.

[34] Kaul DK, Liu XD, Zhang X (2006). Peptides based on *α*V-binding domains of erythrocyte ICAM-4 inhibit sickle red cell-endothelial interactions and vaso-occlusion in the microcirculation. *The American Journal of Physiology*.

[35] Wood KC, Hebbel RP, Granger DN (2005). Endothelial cell NADPH oxidase mediates the cerebral microvascular dysfunction in sickle cell transgenic mice. *FASEB Journal*.

[36] Cheung AT, Miller JW, Craig SM (2010). Comparison of real-time microvascular abnormalities in pediatric and adult sickle cell anemia patients. *The American Journal of Hematology*.

[37] Cheung AT, Miller JW, Miguelino MG (2012). Exchange transfusion therapy and its effects on real-time microcirculation in pediatric sickle cell anemia patients: an intravital microscopy study. *Journal of Pediatric Hematology/Oncology*.

[38] Cheung ATW, Chan MS, Ramanujam S (2004). Effects of poloxamer 188 treatment on sickle cell vaso-occlusive crisis: computer-assisted intravital microscopy study. *Journal of Investigative Medicine*.

[39] Grotta J (2012). Timing of thrombolysis for acute ischemic stroke: “timing is everything” or ‘everyone is different’. *Annals of the New York Academy of Sciences*.

[40] Rutkow IM, Lipton JM (1974). The sickle cell complexity. *The Journal of the American Medical Association*.

[41] Warth JA, Rucknagel DL (1983). The increasing complexity of sickle cell anemia. *Progress in Hematology*.

[42] Frenette PS (2002). Sickle cell vaso-occlusion: multistep and multicellular paradigm. *Current Opinion in Hematology*.

[43] Hebbel RP, Vercellotti GM, Nath KA (2009). A systems biology consideration of the vasculopathy of sickle cell anemia: the need for multi-modality chemo-prophylaxis. *Cardiovascular and Hematological Disorders*.

[44] Castro OL, Gordeuk VR, Gladwin MT, Steinberg MH (2011). Senicapoc trial results support the existence of different sub-phenotypes of sickle cell disease with possible drug-induced phenotypic shifts. *The British Journal of Haematology*.

[45] Moreira LS, de Andrade TG, Albuquerque DM (2008). Identification of differentially expressed genes induced by hydroxyurea in reticulocytes from sickle cell anaemia patients. *Clinical and Experimental Pharmacology and Physiology*.

[46] Rybicki AC, Fabry ME, Does MD, Kaul DK, Nagel RL (2003). Differential gene expression in the kidney of sickle cell transgenic mice: upregulated genes. *Blood Cells, Molecules, and Diseases*.

[47] Milbauer LC, Wei P, Enenstein J (2008). Genetic endothelial systems biology of sickle stroke risk. *Blood*.

[48] Jison ML, Munson PJ, Barb JJ (2004). Blood mononuclear cell gene expression profiles characterize the oxidant, hemolytic, and inflammatory stress of sickle cell disease. *Blood*.

[49] Yuditskaya S, Suffredini AF, Kato GJ (2010). The proteome of sickle cell disease: insights from exploratory proteomic profiling. *Expert Review of Proteomics*.

[50] Sebastiani P, Solovieff N, Hartley SW (2010). Genetic modifiers of the severity of sickle cell anemia identified through a genome-wide association study. *The American Journal of Hematology*.

[51] Walker AL, Steward S, Howard TA (2011). Epigenetic and molecular profiles of erythroid cells after hydroxyurea treatment in sickle cell anemia. *Blood*.

[52] Mitchell M (2009). *Complexity: A Guided Tour*.

[53] Von Bertalanffy L (1950). The theory of open systems in physics and biology. *Science*.

[54] Wright D (2008). *Thinking in Systems: A Primer/ Donella H. Meadows*.

[55] Kitano H (2002). Systems biology: a brief overview. *Science*.

[56] Breitling R (2010). What is systems biology?. *Frontiers in Physiology*.

[57] Machado D, Costa RS, Rocha M, Ferreira EC, Tidor B, Rocha I (2011). Modeling formalisms in systems biology. *AMB Express*.

[58] Joyner MJ (2011). Giant sucking sound: can physiology fill the intellectual void left by the reductionists?. *Journal of Applied Physiology*.

[59] Alon U (2007). *An Introduction to Systems Biology: Design Principles of Biologic Circuits*.

[60] Ofori-Acquah SF, Buchanan ID, Osunkwo I (2012). Elevated circulating angiogenic progenitors and white blood cells are associated with hypoxia-inducible angiogenic growth factors in children with sickle cell disease. *Anemia*.

[61] Buchheit T, van de Ven T, Shaw A (2012). Epigenetics and the transition from acute to chronic pain. *Pain*.

[62] Younger JW, Chu LF, D'Arcy NT, Trott KE, Jastrzab LE, MacKey SC (2011). Prescription opioid analgesics rapidly change the human brain. *Pain*.

[63] Apkarian AV, Sosa Y, Sonty S (2004). Chronic back pain is associated with decreased prefrontal and thalamic gray matter density. *Journal of Neuroscience*.

[64] Hyacinth HI, Gee BE, Adamkiewicz TV (2012). Plasma BDNF and PDGF-AA levels are associated with high TCD velocity and stroke in children with sickle cell anemia. *Cytokine*.

[65] Hyacinth HI, Gee BE, Voeks JH, Adams RJ, Hibbert JM (2012). High frequency RBC transfusion is associated with decreased serum markers of neurodegeneration, vascular remodeling and inflammation. *Blood*.

[66] Kato GJ, Gladwin MT, Steinberg MH (2007). Deconstructing sickle cell disease: reappraisal of the role of hemolysis in the development of clinical subphenotypes. *Blood Reviews*.

[67] Ware RE, Helms RW (2012). Stroke with transfusions changing to hydroxyurea (SWiTCH). *Blood*.

[68] Scothorn DJ, Price C, Schwartz D (2002). Risk of recurrent stroke in children with sickle cell disease receiving blood transfusion therapy for at least five years after initial stroke. *Journal of Pediatrics*.

[69] Embury SH, Matsui NM, Ramanujam S (2004). The contribution of endothelial cell P-selectin to the microvascular flow of mouse sickle erythrocytes in vivo. *Blood*.

[70] Hebbel RP (2008). Adhesion of sickle red cells to endothelium: myths and future directions. *Transfusion Clinique et Biologique*.

[71] Borsook D, Becerra L, Hargreaves R (2011). Biomarkers for chronic pain and analgesia—part 1: the need, reality, challenges, and solutions. *Discovery Medicine*.

[72] Borsook D, Becerra L, Hargreaves R (2011). Biomarkers for chronic pain and analgesia—part 2: how, where, and what to look for using functional imaging. *Discovery Medicine*.

[73] Brown JE, Chatterjee N, Younger J, Mackey S (2011). Towards a physiology-based measure of pain: patterns of human brain activity distinguish painful from non-painful thermal stimulation. *PLoS ONE*.

[74] (2011). *National Institutes of Health Blueprint for Neurosciences Fact Sheet*.

